# A novel case of two siblings harbouring homozygous variant in the *NEUROG1* gene with autism as an additional phenotype: a case report

**DOI:** 10.1186/s12883-023-03065-1

**Published:** 2023-01-16

**Authors:** Frenny Sheth, Jhanvi Shah, Ketan Patel, Darshan Patel, Deepika Jain, Jayesh Sheth, Harsh Sheth

**Affiliations:** 1 FRIGE’s Institute of Human Genetics, FRIGE House, Jodhpur Gam Road, 380015 Satellite, Ahmedabad, India; 2 Speciality Homeopathy Clinic, Ahmedabad, India; 3 Charotar Institute of Paramedical Sciences, Charotar University of Science and Technology, Changa, Gujarat India; 4 Shishu Child Development and Early Intervention Centre, Ahmedabad, India

**Keywords:** Autism spectrum disorder (ASD), Basic helix-loop-helix (bHLH), Congenital cranial dysinnervation disorders (CCDD), Cranial nerves, Loss of function, *NEUROG1* gene, Neurogenin

## Abstract

**Introduction:**

*NEUROG1* gene is yet to be associated with a set of human phenotypes in the OMIM database. Three cases have previously been diagnosed with cranial dysinnervation due to biallelic variants in the *NEUROG1* gene. This is the fourth and a novel report of a sibling pair harboring a homozygous variant in the *NEUROG1* gene with autism as an additional phenotype. A brief review of the literature in conjunction with a genotype–phenotype correlation has been described. A potential hypothesis for the presence of the autistic phenotype in the present case has also been elucidated.

**Case presentation:**

A female aged 6 years and 9 months born to endogamous and phenotypically healthy parents was diagnosed with global developmental delay, autism spectrum disorder, hearing loss, corneal opacity and no eye blinking. Her MRI of the brain revealed mild peritrigonal white matter hyperintensity, and MRI and CT scan of the temporal bones showed abnormal cranial nerves. The proband’s younger sister, aged 4-years, was similarly affected. Whole exome sequencing was performed in the proband, which revealed a novel homozygous, likely pathogenic, truncating frameshift variant, c.228_231dup (p.Thr78ProfsTer122) in exon 1 of the *NEUROG1* gene (ENST00000314744.4). Segregation analysis by Sanger sequencing showed the proband and her younger sister to be homozygotes and their parents to be heterozygous carriers.

**Conclusion:**

This is the fourth report across the globe with a variant identified in the *NEUROG1* gene to be associated with cranial dysinnervation phenotype. An additional phenotype of autism in two female siblings was a novel observation. We provide a hypothetical framework which could explain the pleiotropic effect of a dysfunctional NEUROG1 protein leading to autism and posit it as a candidate for diagnosis of autism spectrum disorder with congenital cranial dysinnervation disorder.

## Background

The congenital cranial dysinnervation disorder (CCDD) is an umbrella term that groups heterogeneous, congenital, non-progressive neurogenic disorders caused by abnormal development of cranial nerves with dysinnervation. These include- Moebius syndrome and its variants, Duane syndrome, congenital fibrosis of external ocular muscles, congenital ptosis, congenital facial palsy and horizontal gaze palsy with progressive scoliosis (1). Neurogenin (NGN) proteins have been associated with cranial dysinnervation. NGNs are a subfamily of group ‘A’ basic helix-loop-helix proteins (bHLH) and class II transcriptional factors (2). The bHLH motif is composed of two highly conserved and specific regions that consists of nearly 60 amino acids, a basic domain that binds to the DNA at a hexanucleotide consensus sequence (i.e., E box—5'-CANNTG-3') and the HLH region that consists of hydrophobic residues that aids in dimerization (3). Binding of the NGN protein to the E-box domain of genes involved in the neurogenesis pathway is critical for regulating their spatio-temporal expression and neuronal differentiation. There are three known NGN proteins in humans- NGN1, NGN2 and NGN3, with NGN1 and 2 being associated with neuronal disorders (4). They are expressed in the placodal ectoderm and regulate a cascade of downstream bHLH proteins leading to the conversion of ectodermal cells to neurons and their differentiation (5). Additionally, they have also been known to regulate astrocyte differentiation by inhibiting gliogenesis (6). During neurogenesis, NGN1 and NGN2 determine the fate of proximal and distal cranial sensory ganglia, respectively (5,7). The proximal ganglia, trigeminal and vestibulocochlear contribute to the cranial nerves V and VIII, respectively (8). In mice, loss of function of the NGN1 protein has been shown to affect cranial ganglia and cranial sensory neurons. These mice showed absence of milk in their stomach, suggesting a suckling defect and died as neonates within 12 h of birth (5). In humans, NGN1 protein is encoded by the *NEUROG1* gene, which is located at 5q31.1 locus. This is a single exon gene, encoding a 237 amino acid long protein. The amino acids 92–144 code for the helix-loop-helix DNA-binding domain of the protein and is suggested to be highly intolerant to variants (9). Three cases have previously been reported with variants in the *NEUROG1* gene that are associated with cranial dysinnervation phenotype (10–12). This article reports the fourth family across the globe consisting of two children (siblings) with a similar phenotype together with autism involving a likely pathogenic variant in the *NEUROG1* gene. This is also the first report to present autism as an additional phenotype being associated with the *NEUROG1* gene.

## Case presentation

**Fig. 1:**
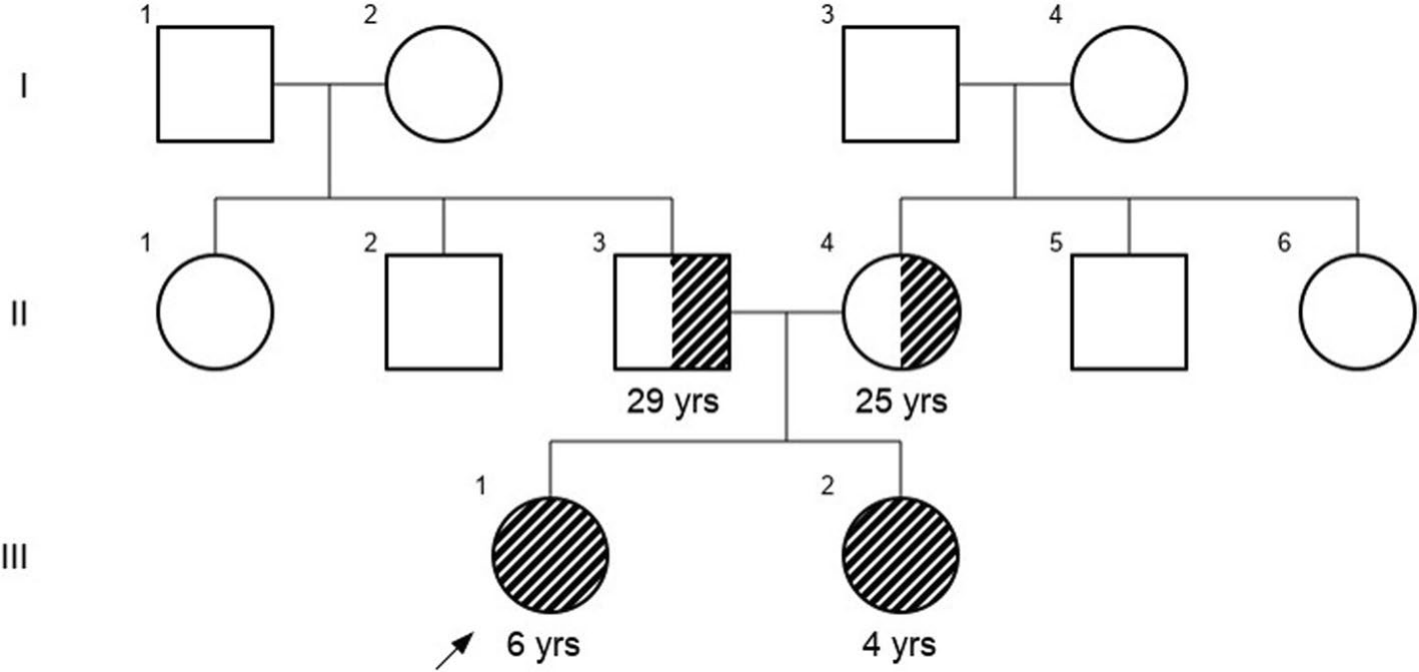
Pedigree chart of the proband’s family. Affected individuals are shown in gray stripes

**Fig. 2:**
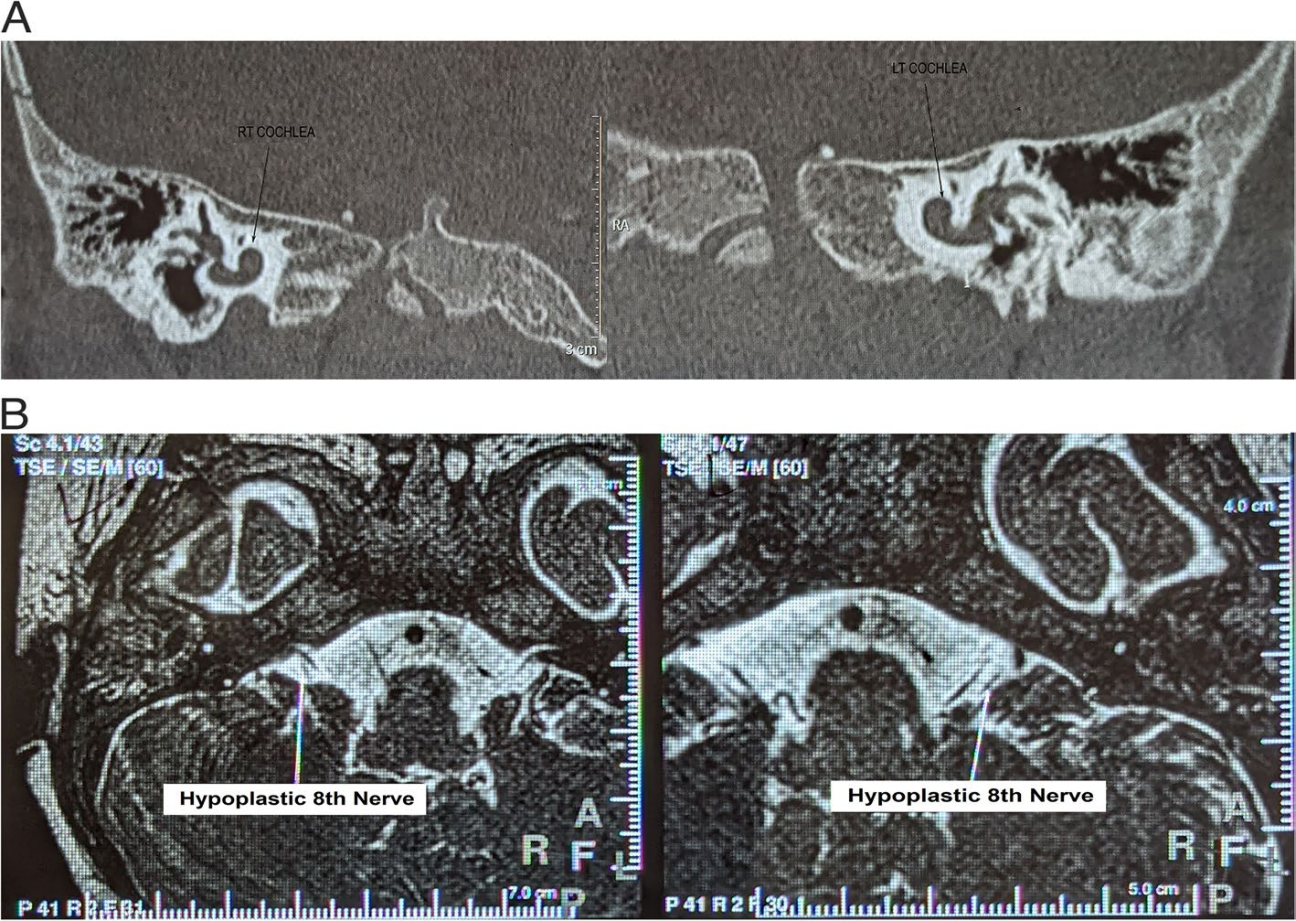
Radiographic images of the inner ear. **A**) Hypoplastic cochlea with presence of only half basal turns on either side in elder sib. **B**) Hypoplastic 8^th^ nerves on both sides

An endogamous couple presented to the clinic with two affected female children of Indian origin. Both sisters were assessed for their clinical manifestations, although initially, only the elder sister underwent a thorough genetic evaluation. The proband (elder sister) was aged six years and nine months at the time of evaluation (Fig. 1). She was born full term by normal delivery. She weighed 3 kg at birth and cried soon thereafter. She was admitted to the NICU in view of high-grade fever at day two of life. She had a history of excessive crying in the first three years of life and had difficulty in chewing solid foods. Profound global developmental delay- attaining sitting at one year six months, standing at five years, walking at five years and six months, and babbling after four years of age was noted. On examination, her head circumference, weight, height and arm span measured 49 cm, 18.7 kg, 115 cm and 114 cm, respectively. All these anthropometric measurements were normal for age (13). Flat occiput, facial asymmetry, mild ptosis, arched eyebrows and long palpebral fissures, an expressionless face, no eye blinking and gait difficulties were noted. She also showed deficits in social communication and interaction, deficits in non-verbal communication along with perseverative interests in certain objects, and stereotypy that included hand flapping and swaying of the body. She was diagnosed with autism spectrum disorder according to the DSM-V criteria (14). Magnetic resonance imaging (MRI) analysis of her brain revealed a mild peritrigonal white matter hyperintensity and MRI of the temporal bones showed bilateral cochlear hypoplasia (Fig. 2A) with the presence of only half basal turns, bilateral hypoplastic eighth nerve (Fig. 2B) and absent modiolus. Furthermore, computerized tomography (CT) scan of the temporal bones showed hypoplastic cochlea with presence of only half basal turns bilaterally, bilateral narrowing of internal auditory canals and dilated vestibules. Ophthalmic evaluation reported decreased corneal opacity in the right eye as compared to dense corneal opacity with dryness in the left eye.

The younger sister, aged 4-years portrayed a similar but less severe phenotype. Whilst the craniofacial features were similar to that of her elder sister, she did not have difficulty in chewing food. Her developmental milestones were delayed but were attained sooner than her sister. Her ophthalmic evaluation reported congenital corneal anesthesia syndrome with bilateral neurotrophic ulcer. Her MRI and CT scan analysis of the temporal bones showed cochlear hypoplasia of one and a half turns with stenotic internal auditory canal and absent vestibulo-cochlear nerve on both sides. Changes of otitis media with mastoiditis could also be visualised, suggesting a recent ear infection.

The entire genetic evaluation was conducted for the elder sister, subsequent to receiving the institutional ethics committee approval as per the Helsinki declaration and written informed consent from the parents. Karyotype was first performed followed by SNP oligonucleotide microarray and whole exome sequencing (WES). Karyotype by GTG banding at 500 bands resolution showed a normal chromosome constitution (46,XX). Microarray performed on the Affymetrix platform using the CytoSCan Optima array (Thermo Fisher, USA) and its analysis using Chromosome Analysis Suite software was carried out according to the manufacturer’s protocol, and did not show any cryptic genomic imbalance. However, a copy neutral loss of heterozygosity (cnLOH) of approximately 7% was observed in the autosomal region which included a 30 Mb stretch from 5q23.1 to 5q32. WES was performed using the Agilent SureSelect v6 enrichment kit (Agilent, USA) and Illumina HiSeq platform (Illumina, USA) at an average read depth of approximately 100x. Fastq files from the sequencing machine were aligned against the human reference genome (GRCh37/hg19) using BWA (15) and SNVs and indels were called using GATK v4.1 using GATK’s best practice guidelines (16). Variant annotation and prioritization was carried out using JANNOVAR and Exomiser v12.1.0 (17,18) with the available phenotype information translated into standardized human phenotype ontologies.

**Fig. 3:**
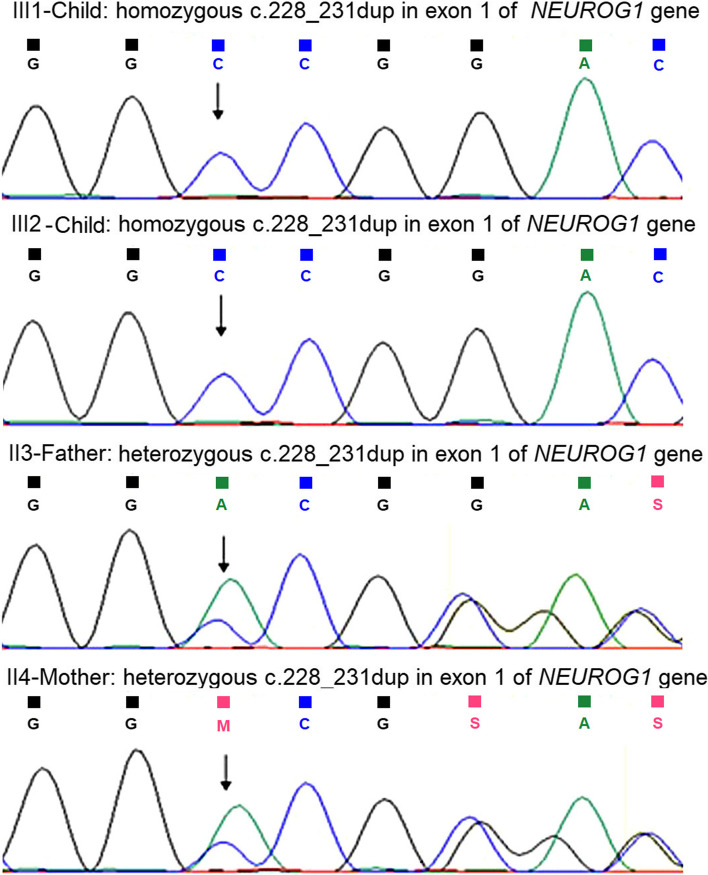
Sanger sequencing chromatogram images of the siblings and their parents indicating homozygosity and heterozygosity, respectively for the 4 bp insertion

WES identified a novel, homozygous four base pair duplication c.228_231dup in exon 1 of the *NEUROG1* gene that results in a frameshift and premature truncation of the protein 122 amino acids downstream to codon 78 (p.Thr78Profs*122; ENST00000314744.4; ClinVar ID: SCV001960904.1). This variant was not observed in the 1000 genomes and gnomAD databases and the *in-silico* predictions that the variant is disease causing by MutationTaster2 (19) and MetaDome showed the region to be slightly intolerant to variation with a score of 0.6 (9). Validation of the variant and parental segregation analysis by Sanger sequencing showed the variant to be present in the child as well as in her affected younger sister in the homozygous state, and the parents to be heterozygous carriers, suggesting an autosomal recessive mode of inheritance (Fig. 3). Based on the above evidence, the variant was classified as likely pathogenic according to the ACMG-AMP classification system (20) and ClinGen framework (21) considering the following criteria: PM2 (supporting), PP3 (supporting), PP4 (supporting), PP1 (strong) and PVS1 (strong).

## Discussion and conclusion

At present, the *NEUROG1* gene does not have a corresponding phenotype in the OMIM database. However, three cases have previously been reported with variants in this gene from all around the globe and to the best of our knowledge, this would be the fourth report. The *NEUROG1* gene was for the first time associated with a disease phenotype in 2013 in a six year old male child of Turkish origin with a 115 kb homozygous deletion encompassing the entire *NEUROG1* gene (10). Thereafter in 2015, a 12 year old male child of Middle Eastern origin with clinical findings of corneal opacity, mild intellectual disability and absent corneal reflex was identified with a homozygous missense variant c.347G > T (p.Arg116Leu) in the *NEUROG1* gene using clinical exome sequencing (11). The third report was of a 12 year old male proband of Portuguese ethnicity who was identified with a homozygous nonsense variant c.202G > T (p.Glu68Ter) (12). The present report discusses a sibling pair harbouring a frameshift variant in the *NEUROG1* gene as a potential cause of their phenotype similar to those observed in the previous three cases.

The major clinical finding of all reported patients to date includes developmental delay, corneal opacity along with absent corneal reflex, plagiocephaly, expressionless face with asymmetry, bilateral hearing loss, and intellectual disability. The major neuroimaging findings described thus far include agenesis or dysgenesis of the VIII cranial nerve and cochlear malformations. These features were a result of maldevelopment of the sensory cranial ganglia that were akin to those observed in the *Ngn1* gene knockout mice. The knockout mice that lacked the NGN1 protein showed defects in the midbrain, dorsal root ganglia and certain cranial sensory ganglia, in particular, the trigeminal and vestibulo-cochlear ganglia (5). Truncation of cranial nerve V and absence of cranial nerve VIII were observed in these mutant mice (5). Similarly in humans, the trigeminal placode gives rise to the trigeminal nerve/ cranial nerve V that contributes to the innervation of the face. Dysinnervation of this nerve may likely result in atypical facial features. Likewise, dysinnervation of the cranial nerve VIII/ vestibular and cochlear nerve, that arises from the otic placode and contributes to equilibrium, eye movements and hearing, respectively, would result in balance issues, atypical ophthalmic findings and inner ear malformations. All of these findings have been reported in patients with variants in the *NEUROG1* gene. Nonetheless, the phenotype as a result of the dysinnervation of the vestibulocochlear nerve was more noticeable, whereas the dysinnervation of the cranial nerve V has only been reported in one patient (12). In contrast, both sisters in the present case did not present with any abnormalities of the Vth cranial nerve. However, the elder sister showed only half basal turns of the cochlea whereas the younger sister showed one and a half basal turns. This is the first time that a half basal turn has been observed in a patient with a variant in the *NEUROG1* gene. This could also be the result of a more severe phenotype in the elder sister compared to all other patients including her younger sister. Considering that all the manifestations were a result of aberrant sensory cranial nerves, the syndrome has been classified as CCDD and genetic variants in the *NEUROG1* gene as a potential cause of it.

Autism was another clinical finding in the present case that was not reported in the probands of the previous three cases. Autism spectrum disorder (ASD) is a complex neurodevelopmental condition whereby, an affected individual is primarily diagnosed with deficit in social interactions and stereotypic behavior (22). This could lead to a lifelong condition that negatively impacts social functioning and quality of life in affected individuals. Whilst the prior three published cases presented with developmental delay/ intellectual disability, no further clinical details were provided that could help towards post hoc differential diagnosis of ASD. Therefore, it is plausible that prior studies could have missed ASD as a phenotype. Recent research suggests that dysregulation of cellular processes such as neurogenesis could be a potential underlying cause of autism (23). Sensory cranial nerves are formed through the process of neurogenesis and *NEUROG1* is one such gene that regulates this process. Hence, it is possible that the autistic phenotype in humans could occur due to dysregulation of this gene that leads to the formation of abnormal sensory cranial nerves.

**Fig. 4:**
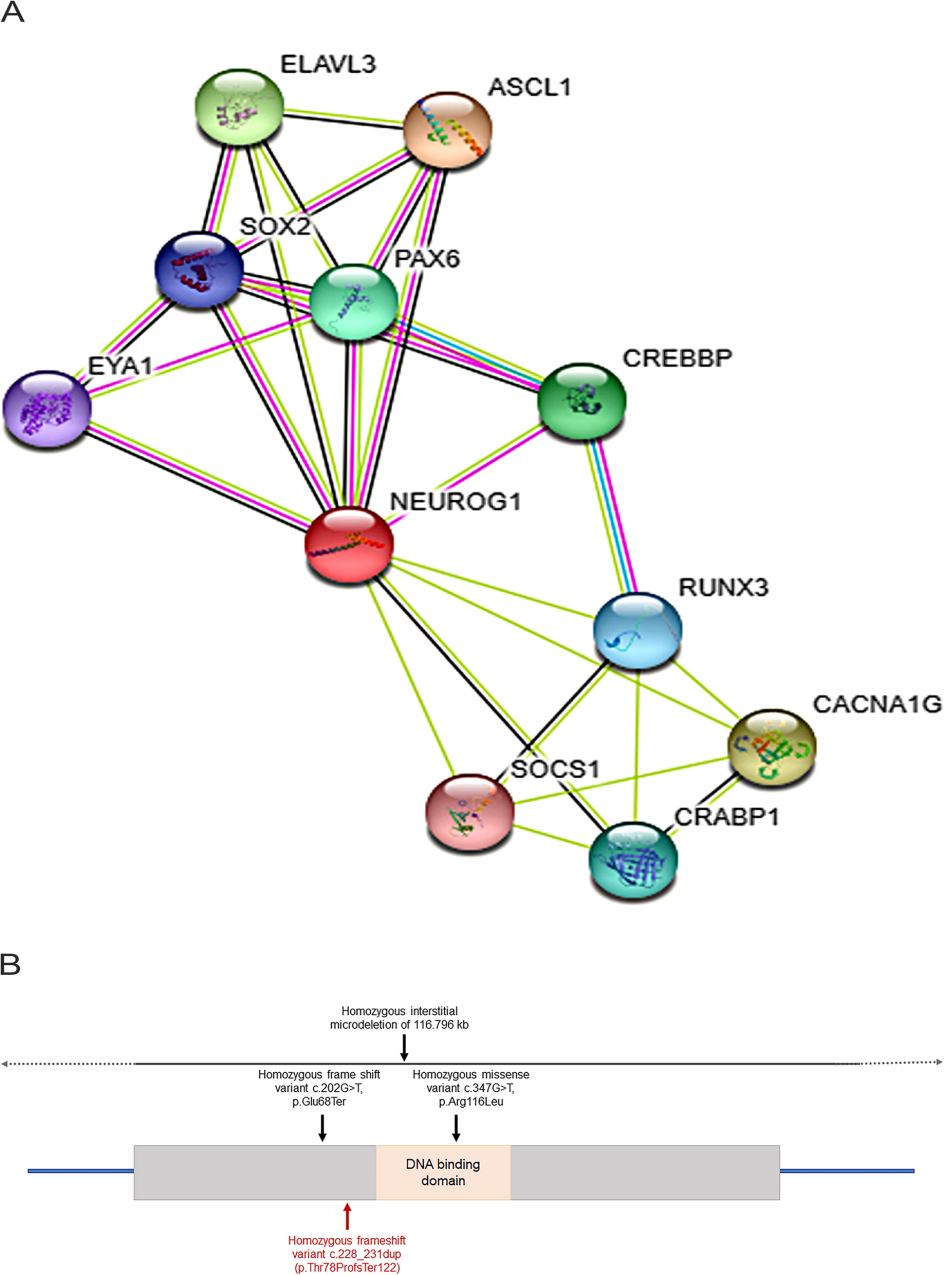
Schematic representation of (A) STRING interaction network of the NEUROG1 protein. (B) *NEUROG1* gene along with the corresponding domains and variants observed till date. Variant highlighted in red corresponds to the present case. Solid line extending with dotted arrows indicates the large deletion encompassing the *NEUROG1* gene and beyond

Additionally, linkage studies have also shown the *NEUROG1* gene to be associated with schizophrenia (24). Further study suggested that transcriptional activity of the NEUROG1 protein could affect expression of multiple downstream genes and cause a pleiotropic effect leading to schizophrenia (24). Likewise, it can be postulated that autism also being a behavioral phenotype with a complex genetic etiology could be a result of this pleiotropic effect. STRING analysis suggests that NEUROG1 protein interacts with CREBBP and PAX6 proteins and is found to be co-expressed with ELAVL3 protein in humans (Fig. 4A) (25). *CREBBP, PAX6* and *ELAVL3* genes are associated with autism spectrum disorder phenotype with high confidence according to the SFARI Gene database (26). Therefore, it is plausible that a disrupted function of the NEUROG1 protein could lead to an ASD phenotype, however, this would require further studies.

All the three published cases and the sibling pair in the present study were identified with a homozygous variant in the *NEUROG1* gene (10,12). These variants included a microdeletion of ~ 115 kb encompassing the entire *NEUROG1* gene (10), a missense variant c.347G > T (p.Arg116Leu) (11), a nonsense variant c.202G > T (p.Glu68Ter) (12) and a frameshift variant c.228_231dup (p.Thr78ProfsTer122) detected in the present case (Fig. 4B). Despite the variant type, all were found to disrupt the bHLH domain of the NGN1 protein. In the case that harboured an entire gene deletion, no protein was produced, and hence there was no bHLH domain to function. The missense variant in the previously reported case lies within the bHLH domain and is proposed to result in the structural change of the protein. The truncating nonsense and frameshift variants were present before or in the bHLH domain leading to an immature stop codon or changing the coding frame resulting in a different amino acid sequence, respectively.

Interestingly, it is now suggested that not all truncating variants undergo nonsense-mediated decay (NMD) (27). Indeed, genomic analysis shows that in approximately 36% of the genes, > 75% of the coding sequence would allow putative loss of function (pLOF) variants/ protein truncating variants to atleast partially evade NMD (27). The NMD detector model developed by Lindeboom et al*.*, predicts that the pLOF variant detected in our case would most likely lead to evasion of NMD (NMD score = 0.07) (28). In such scenarios, where a transcription factor is the primary protein that regulates downstream genes-proteins would potentially result in phenotypic pleiotropy. Focusing on the frameshift variant observed in the present case, it is possible that an abnormal protein was formed rather than undergoing NMD that could interact with genes downstream to *NEUROG1*. This could be the pathogenesis of ASD observed in the two sisters in the present report. Whilst analysis of the NEUROG1 protein expression using Western blot could have helped to test this hypothesis, the patient’s guardians were lost to follow-up and re-sampling could not been carried out for this analysis. This is a limitation of the current study. With a collective data obtained from the functional study of the *NEUROG1* gene, a striking resemblance in the phenotypes of the present case with those reported previously and their concordance with the phenotypes observed in *Ngn1* knockout mice, the *NEUROG1* gene is proposed to be causative of the CCDD. In conclusion, a consistent inheritance pattern encompassing all variants in the form of homozygosity affecting both males and females further supports an autosomal recessive inheritance that can be linked to the *NEUROG1* gene. Variation in the *NEUROG1* gene can be associated with two additional features, namely a behavioral phenotype in the form of autism and the presence of a more severe structural deformation form of the cochlea having only half basal turns. Otitis media in the younger sister could also be considered as a novel phenotype that could be due to inner ear malformations. Nonetheless, a hypothesis of the abnormal protein formed due to the frameshift variant that escapes NMD could cause a pleiotropic effect leading to autism, makes NEUROG1 a potential novel candidate for the diagnosis of syndromic ASD with CCDD.

## Data Availability

Raw data of the patient (EGA patient ID: EGAN00003502459) for whole exome sequencing (EGA Study ID: EGAS00001006060) and chromosomal microarray (EGA Study ID: EGAS00001006439) are available on the EGA website (European Genome-Phenome Archive) under the title “Genetic architecture of autism spectrum disorders in India”.

## Abbreviations

CCDD: Congenital cranial dysinnervation disorders
bHLH: Basic helix-loop-helix
NGN: Neurogenin
WES: Whole exome sequencing
ASD: Autism spectrum disorder
DSM-V: Diagnostic and Statistical Manual of Mental Disorders – 5
MRI: Magnetic Resonance Imaging
CT: Computed Tomography
OMIM: Online Mendelian Inheritance in Man
ChAS: Chromosome Analysis Suite
CMA: Chromosomal Microarray
NMD: Nonsense Mediated Decay
